# BG-flow, a new flow cytometry tool for G-quadruplex quantification in fixed cells

**DOI:** 10.1186/s12915-021-00986-6

**Published:** 2021-03-11

**Authors:** Alessio De Magis, Melanie Kastl, Peter Brossart, Annkristin Heine, Katrin Paeschke

**Affiliations:** Department of Oncology, Hematology and Rheumatology, University Hospital Bonn, Venusberg-Campus 1, 53127 Bonn, Germany

**Keywords:** Secondary DNA structures, G-quadruplex, Flow cytometry

## Abstract

**Background:**

Nucleic acids can fold into non-canonical secondary structures named G-quadruplexes (G4s), which consist of guanine-rich sequences stacked into guanine tetrads stabilized by Hoogsteen hydrogen bonding, π-π interactions, and monovalent cations. G4 structure formation and properties are well established in vitro, but potential in vivo functions remain controversial. G4s are evolutionarily enriched at distinct, functional genomic loci, and both genetic and molecular findings indicate that G4s are involved in multiple aspects of cellular homeostasis. In order to gain a deeper understanding of the function of G4 structures and the trigger signals for their formation, robust biochemical methods are needed to detect and quantify G4 structures in living cells. Currently available methods mostly rely on fluorescence microscopy or deep sequencing of immunoprecipitated DNA or RNA using G4-specific antibodies. These methods provide a clear picture of the cellular or genomic localization of G4 structures but are very time-consuming. Here, we assembled a novel protocol that uses the G4-specific antibody BG4 to quantify G4 structures by flow cytometry (BG-flow).

**Results:**

We describe and validate a flow cytometry-based protocol for quantifying G4 levels by using the G4-specific antibody BG4 to label standard cultured cells (Hela and THP-1) as well as primary cells obtained from human blood (peripheral blood mononuclear cells (PBMCs)). We additionally determined changes in G4 levels during the cell cycle in immortalized MCF-7 cells, and validated changes previously observed in G4 levels by treating mouse macrophages with the G4-stabilizing agent pyridostatin (PDS).

**Conclusion:**

We provide mechanistic proof that BG-flow is working in different kinds of cells ranging from mouse to humans. We propose that BG-flow can be combined with additional antibodies for cell surface markers to determine G4 structures in subpopulations of cells, which will be beneficial to address the relevance and consequences of G4 structures in mixed cell populations. This will support ongoing research that discusses G4 structures as a novel diagnostic tool.

**Supplementary Information:**

The online version contains supplementary material available at 10.1186/s12915-021-00986-6.

## Background

The great structural polymorphism of nucleic acids enables a plethora of secondary and tertiary structures that add up extra layers of genetic information other than the “simple” primary sequence alone. Among these, a particular type of non-canonical DNA/RNA secondary structure, named G-quadruplex (G4), folds from guanine-rich sequences (G4 motifs) into (at least two) stacked guanine tetrads stabilized by Hoogsteen hydrogen bonding, π-π stacking interactions, and monovalent cations [1]. Although the actual formation and function of G4 structures in vivo has been long debated, there is now cogent evidence of their presence and function in living cells [2]. G4 motifs are conserved throughout evolution. The human genome alone presents 700,000 potential G4-forming regions significantly enriched in key genomic sites such as telomeres, promoters, splicing sites, origins of replication, and immunoglobulin switch regions [3–6].

The visualization and quantification of G4 structures in cells vastly relies on chromatin immunoprecipitation coupled to sequencing (ChIP-seq) or immunofluorescence (IF) by means of G4-specific single-chain antibodies (BG4, D1) [7, 8]. Although very informative at different levels, both approaches still come with some drawbacks. ChIP-seq is time-consuming and costly and although it provides sequence-level information, this result is averaged over a cell population. IF can provide single-cell information, but requires relatively long data acquisition and processing time and can be challenging in the case of cells in suspension or that hardly attach to a slide.

A very interesting addition to the currently available techniques to detect, visualize, and quantitatively analyze G4 structures is flow cytometry (FC), which can provide multiparametric data on large cell populations [9]. FC bears considerable advantages by providing detailed, population-scale quantitative insights and can also identify population subsets with fairly fast data acquisition.

Here, we describe and validate a fast and reliable FC-based protocol for quantifying G4 levels in total cells using the widely tested and validated G4-specific antibody BG4. IF and ChIP-seq experiments using BG4 revealed that G4 structures form in different cells at specific regions and are enriched in cancer cells [7, 8, 10, 11] where they contribute, at least in breast cancer, to tumor subtype generation [12]. With this new protocol (BG-flow), G4 structures can be analyzed fast and reliable throughout the cell cycle in different cell lines or from cells isolated from blood or tissue samples. We confirmed G4 levels with published values from the literature as well as data obtained by microscopic imaging methods.

## Results

### Flow cytometry efficiently detects ligand-induced G4 changes in HeLa cell

**Fig. 1:**
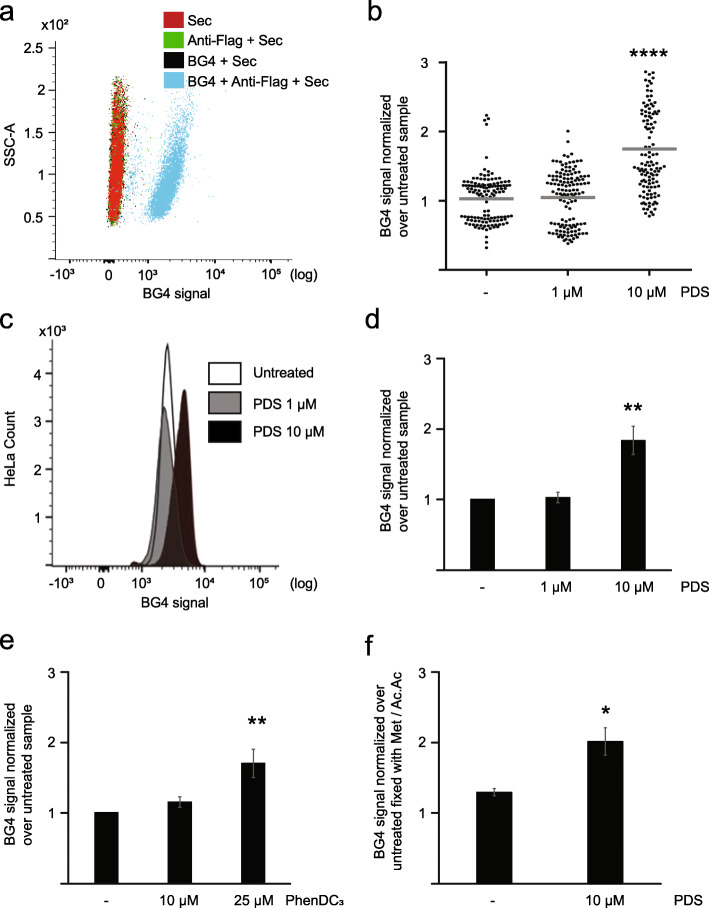
PDS treatment induced an increased BG4 signal in HeLa cells. **a** Histogram plot of the BG4 signal distribution in HeLa cells, incubated with BG4 + secondary (black), anti-flag + secondary (green), and only secondary antibody (red). The untreated cell population is depicted in blue. **b** G4 levels determined by fluorescence intensity of nuclei normalized over WT. Results are based on the average of *n* = 3 biologically independent experiments. The horizontal gray line represents the mean value. Significance was determined using an ordinary one-way ANOVA multiple comparison test. Asterisks indicate statistical significance in comparison with wildtype cells: ****p < 0.0001. **c** Histogram plot of the BG4 signal distribution in untreated HeLa cells (white) and cells incubated 24 h with 1 μM (gray) or 10 μM (black) PDS. **d** Quantification of the BG4 signal in untreated HeLa cells and cells incubated 24 h with 1 μM or 10 μM PDS. Plotted results were based on the average of at least *n* = 3 biologically independent experiments. Significance was calculated based on a one-sided Student’s *t*-test. Asterisks indicate statistical significance in comparison with wildtype cells: **p < 0.01. **e** Quantification of the BG4 signal in untreated HeLa cells and cells incubated 24 h with 10 μM or 25 μM PhenDC_3_. Results are based on the average of *n* = 3 biologically independent experiments. Significance was calculated based on a one-sided Student’s *t*-test. Asterisks indicate statistical significance in comparison with wildtype cells: **p < 0.01. **f** Quantification of the BG4 signal in untreated HeLa cells and cells incubated 24 h with 10 μM PDS and fixed with PFA. Plotted results were based on the average of *n* = 3 biologically independent experiments. Significance was calculated based on a one-sided Student’s *t*-test. Asterisks indicate statistical significance in comparison with wildtype cells: *p < 0.05. An excel file providing individual data values is available in the section “Availability of data and materials”

We have created a new FC-based approach to quantitatively measure G4 structure levels in cells (BG-flow). To validate this protocol, we measured if and how the FC signal is altered in cells incubated either with or without BG4 antibody (Fig. 1a). Cells were gated for size (forward scatter (FSC)) and granularity (side scatter (SSC)) as shown in Additional file 1: Fig.S1a. We detected that 81.4% of cells were positive for the BG4 signal. These analyses revealed a clear shift (based on mean fluorescence intensity (MFI)) of the BG4 signal (channel FL1) in comparison to the no BG4 controls, indicating that the antibody detected G4 structures in the cells. BG4 detection in IF as well as in FC was done with three antibodies. First, cells were incubated with BG4, then with anti-FLAG followed by a secondary antibody that harbored the fluorescent signal. Note, BG4 exhibits a FLAG epitope tag. In order to exclude false positive due to unspecific staining of these antibodies, we performed three additional controls: anti-FLAG + secondary antibody, BG4 + secondary, and only secondary antibody (Fig. 1a). Due to the nature of the BG4 antibody (a single-chain antibody), no isotype control is possible. These analyses already provided promising data that G4 structures can be detected by FC.

As a first benchmark for the capability of FC to detect G4s, we chose to compare its performance to the well-established IF detection of G4 structures in HeLa cells upon treatment with the commercially available G4-stabilizing ligand pyridostatin (PDS) [8, 11, 13, 14]. By IF, 10 μM PDS led to a 2.1-fold increase in G4 levels without affecting cell viability [11]. We repeated the published IF staining of HeLa cells that were incubated with either 1 μM or 10 μM PDS for 24 h. Similar to published data [11], 1 μM PDS caused no changes in G4 structure levels, whereas 10 μM resulted in a 1.75-fold increase in G4 structure abundance in comparison to untreated cells (Fig. 1b, Additional file 1: Fig. S1b). Note, in the IF, only the BG4 signal in the nucleus of the cells was quantified. The BG4 signal in the cytoplasm was very low and did not quantitatively alter G4 structure levels. To confirm the flexibility and robustness of the method, we addressed if FC can be used, similar to IF, to detect changes in G4 structure levels after stabilization with PDS. We treated cells with 1 μM and 10 μM of PDS (24 h) and analyzed G4 structure levels by FC. In agreement with the IF data (Additional file 1: Fig. S1a), FC analysis revealed no difference in BG4 signal after incubation with 1 μM of PDS but a clear shift in the histogram pattern after incubation with10 μM PDS (Fig. 1c, Additional file 1: Fig. S1c). Cells were gated for the size (FSC) and granularity (SSC) (Additional file 1: Fig.S1b). The quantification of the BG4 signal showed no fold changes compared to untreated cells for incubation with 1 μM PDS and a 1.8-fold increase for 10 μM PDS (Fig. 1d). These results were in agreement with results obtained by the established BG4 IF protocol, supporting the finding that FC analysis is a valid method to quantify G4 structures in fixed cells.

In order to exclude a bias due to PDS emission in FC, we expanded the analysis to another compound, PhenDC_3_. PhenDC_3_ is a bisquinolinium derivate that has a high affinity for G4 structures and stabilizes them in vitro and in vivo [15]. Two concentrations, 10 and 25 μM, were tested. Our analyses revealed an increase (based on MFI) of the BG4 signal (1.7-fold) in cell incubated with 25 μM PhenDC_3_ compared to untreated HeLa cells (Fig. 1e, Additional file 1: Fig. S1d). No changes were detected with 10 μM PhenDC_3_ (Fig. 1e, Additional file 1: Fig. S1d).

Methanol/acetic acid was used for the fixation of the cells both in IF and FC. Fixation with organic solvents, such as methanol/acetic acid, causes a severe loss of cell membrane integrity and cytoplasmic structures and the consequent loss of RNA G4 structures. In order to understand which is the contribution of cytoplasmatic RNA G4 structures to the overall G4 landscape, we performed the same analysis with paraformaldehyde (PFA) fixation. Both fixation methods have been used for IF [8, 11]. Untreated and PDS-treated cells (10 μM) were fixed with PFA (2% (v/v) in 1 × PBS for 15 min) and G4 structure levels were measured by FC (Fig. 1f, Additional file 1: Fig. S1e). Similar to methanol staining, we revealed a 1.6-fold increase of G4 structures after PDS incubation compared to untreated. Direct comparison showed a marginal increase (1.2-fold) after PFA fixation compared to methanol fixation (Fig. 1f, Additional file 1: Fig. S1e). This data indicated that a minor fraction of cytoplasmic G4 structures was lost due to the fixation with methanol/acetic acid. This change indicates that both fixation methods are working in BG-flow. Further, with methanol fixation, nuclear G4s are quantified, whereas with PFA both nuclear and cytoplasmic G4s are detectable. In the subsequent analysis, we performed methanol/acetic acid fixation, which allowed us to better compare the data to the IF where only the nuclear fraction was quantified (data not shown). Taken together, these results confirm the robustness of the BG-flow technique.

### BG-flow can monitor G4 signal in THP-1 cells

**Fig. 2:**
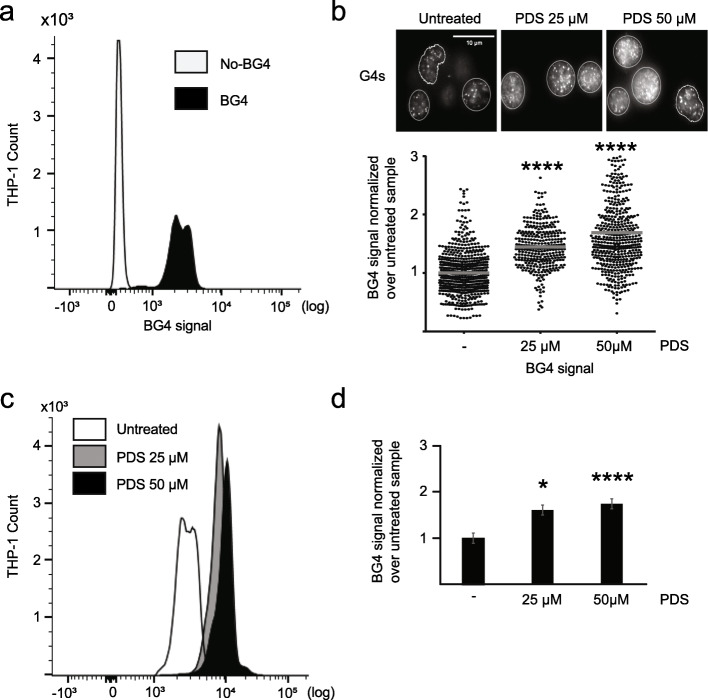
PDS treatment induced an increased BG4 signal in THP-1 cells. **a** Histogram plot of the BG4 signal distribution in THP-1 cells incubated with BG4 (black) or without antibody (white). **b** Microscopic images of IF using BG4 in untreated THP-1 cells and cells incubated 24 h with 25 μM or 50 μM PDS with the BG4 antibody (white signals). Nucleus border is defined by white borders. Scale bar, 10 μm. Below G4 levels are plotted as determined by fluorescence intensity of nuclei normalized over untreated cells. Results are based on the average of at least *n* = 3 biologically independent experiments. The horizontal gray line represents the mean value. Significance was determined using an ordinary one-way ANOVA multiple comparison (****p < 0.0001). **c** Histogram plot of the BG4 signal distribution in untreated THP-1 cells (white) or cells incubated 24 h with 25 μM (gray) or 50 μM (black) PDS. **d** Quantification of the BG4 signal distribution in untreated THP-1 cells and cells incubated 24 h with 25 μM or 50 μM PDS. Results are based on the average of at least *n* = 3 biologically independent experiments. Significance was calculated based on a one-sided Student’s *t*-test. Asterisks indicate statistical significance in comparison with wildtype cells: *p < 0.05, ****p < 0.0001. An excel file providing individual data values is available in the section “Availability of data and materials”

To further validate and extend the working spectrum of BG-flow, we extended the analysis to THP-1 cells. THP-1 cells are human monocytes derived from a patient with acute monocytic leukemia. THP-1 are cells growing in suspension and often used as an in vitro cancer cell model [16], as well as a model to study the monocyte-macrophage differentiation process [17]. Similar to HeLa cells, incubation with BG4 revealed a clear shift (based on MFI) of the BG4 signal in comparison to the antibody-free control. This demonstrated that BG4 detected G4 structures in THP-1 cells (Fig. 2a, Additional file 2: Fig. S2a).

Because the effect of G4 structure stabilization by PDS has not been studied in THP-1 cells, yet, we first determined the cytotoxicity of PDS with MTT assay. We aimed to obtain a survival rate of at least 80% after 24 h to avoid measuring apoptosis/necrosis mechanisms, which likely involve G4 structures. We initially used 25 μM and 50 μM PDS (Additional file 2: Fig. S2b). THP-1 cells were incubated with PDS for 24 h and G4 structure levels were measured by FC and IF (see the “Methods” section). Similar to data from HeLa cells, the two approaches yielded very consistent and reproducible results. Using untreated cells as a reference, G4 structure level changed 1.5- and 1.6-folds (at 25 μM PDS) for IF and FC, respectively, and 1.7-fold for both at 50 μM PDS (Fig. 2b–d, Additional file 2: Fig.S2a, c). As seen with HeLa cells, only the nuclei were used for IF quantification because the signal from the cytoplasm was negligible (data not shown). These results clearly support the finding that BG-flow can be used to measure G4 structure levels also in suspension cells. We also demonstrated for the first time that G4 structures form in human monocytes and that PDS leads to an accumulation of G4 structures in these cells.

### BG-flow can be used to determine cell cycle changes of G4 structures

**Fig. 3.**
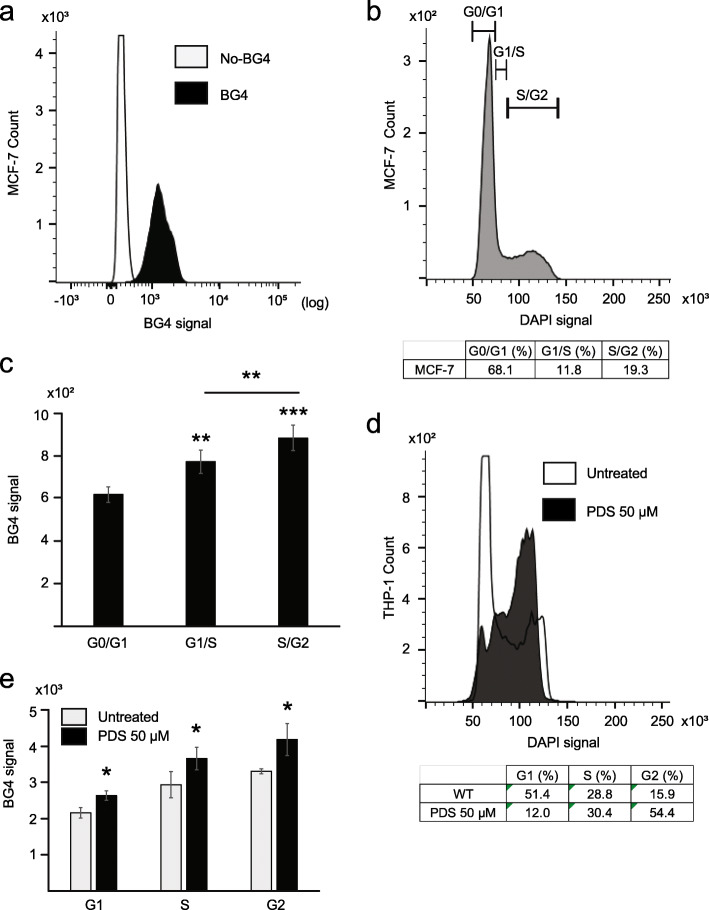
BG-flow is suitable to detect BG4 signal changes in different cell cycle phases. **a** Histogram plot of the BG4 signal distribution in MCF-7 cells incubated with BG4 (black) or without antibody (white). **b** Histogram plot of the DAPI signal in MCF-7 cells. The cell cycle distribution was obtained dividing the cells per DAPI amount. The table states the quantification (% of total) of the cells in the three cell cycle phases. **c** Quantification of the BG4 signal distribution in MCF-7 cells divided per cell cycle phases. Results are based on the average of *n* = 3 biologically independent experiments. Significance was calculated based on the one-sided Student’s *t*-test. Asterisks indicate statistical significance in comparison with wildtype cells: **p < 0.01, ***p < 0.001. **d** Histogram plot of the DAPI signal in untreated THP-1 cells (white) and cells incubated 24 h with 50 μM PDS (black). The table states the cell cycle distribution obtained by analysis with the cell cycle tool in FlowJo. **e** Quantification of the FL1-BG4 signal distribution in untreated THP-1 cells (white) and cells incubated 24 h with 50 μM PDS (black) in the different cell cycle phase. The table states the BG4 signal increase in the different cell cycle phases. Results are based on the average of *n* = 3 biologically independent experiments. An excel file providing individual data values is available in the section “Availability of data and materials”

We wanted to investigate, if BG-flow can be used to determine changes of G4 structure levels throughout the cell cycle in human cells as reported for IF [8]. We co-stained cells with BG4 and 4′,6-diamidino-2-phenylindole (DAPI). DAPI has been used before in FC to determine cell cycle phases [18]. MCF-7, an immortalized breast cancer cell line, was selected due to published data [8]. Similar to HeLa cells, the incubation with BG4 revealed a clear shift (based on MFI) of the BG4 signal in comparison to the antibody-free control. This indicated that BG4 detected G4 structures in MCF-7 cells (Fig. 3a, Additional file 3: Fig.S3a-b). By using the multidimensional cell cycle analysis package in FlowJo [19], we could discriminate each phase of the cell cycle based on the amount of DNA present in the cells indicated by the DAPI signal strength. Three different cell cycle phases were detectable: G0/G1, quiescent cells; G1/S, cells prone to enter in the replication phase; and S/G2, replicative cells (Fig. 2b). G4 structure levels were measured for each MCF-7 cell population and sorted by cell cycle phase. These data showed the highest levels of G4 structures in S/G2 phase (Fig. 3c): 1.5-fold more G4 structures than during G0/G1 phase (Fig. 2c). Here, we confirmed that in MCF-7 cells the cellular G4 landscape substantially varies throughout the cell cycle progression [8]. With minimal G4 structure levels during G0/G1 phase and a strong correlation between maximal G4 structure levels in the S/G2 phase [8, 20]. We next wanted to test if BG-flow can be used to determine G4 structure changes during the cell cycle in THP-1 cells. We co-stained THP-1 cells with BG4 and DAPI. G4 levels were then measured for each THP-1 cell population sorted by cell cycle phase. THP-1 cells showed higher G4 structure levels in both untreated and PDS-treated cells in the G2 phase (Fig. 3e). Note, upon PDS treatment, the increase of the BG4 signal was the same (~ 1.6-fold), regardless of the cell cycle phase (Fig. 3e). Upon PDS treatment, the G2 phase fraction strongly increased (from 15.9 to 54.4%) along with a comparable decrease of the G1 phase population from 51.4 to 12% (Fig. 3d). This data demonstrated that BG-flow can be used in double staining, to determine G4 structure levels during different cell cycle phases. In addition, we demonstrated that in THP-1 cells G4 structure levels peak in the G2 phase. It is not clear why G4 structure levels peak in the G2 phase in THP-1 cells. It could be that THP-1 cells, which are monocytic cells, require G4 structure formation during the G2 phase for a monocytic-specific function. In general, monocytes do not proliferate, are extremely sensitive to reactive oxygen species (ROS), and lack a functional base excision repair and DNA double-strand break repair via nonhomologous end joining [21]. In the G2 phase, the replicated DNA is not yet condensed and DNA is repaired mainly by homologous recombination [22]. One hypothesis is that G4 structures perform a function in the response to ROS in THP-1 cells. G4 structures could also support the differentiation process of monocytes into macrophages or dendritic cells. From the presented data, the function of G4 structures during the G2 phase cannot be explained, but the finding is of great interest and will be further studied.

### BG-flow can be used to determine G4 structure levels in blood cells

**Fig. 4.**
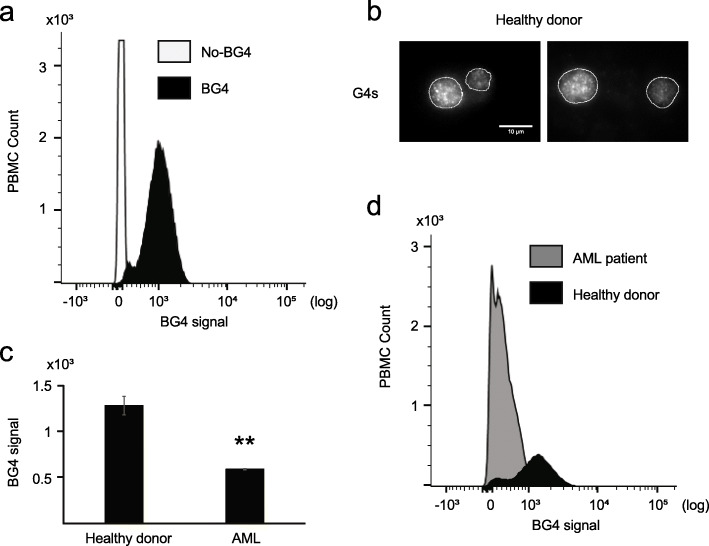
G4 flow cytometry analysis of human PBMC cells. **a** Histogram plot of the BG4 signal distribution in human PBMC incubated with BG4 (black) or without antibody (white). **b** Labeling of PBMC cells from a healthy donor with the BG4 antibody (white). Nucleus border is defined by the white line. Scale bar 10 μm. **c** Quantification of the BG4 signal in human PBMC cells from an AML patient or a healthy donor. Plotted results were based on the average of *n* = 3 technical independent experiments processed at three different time points. Significance was calculated based on a one-sided Student’s *t*-test. Asterisks indicate statistical significance in comparison with wildtype cells: **p < 0.01. **d** Histogram plot of the BG4 signal distribution in human PBMC extract from an AML patient (gray) or a healthy donor (black). An excel file providing individual data values is available in the section “Availability of data and materials”

Next, we tested whether BG-flow can measure G4 structure levels in peripheral blood mononuclear cells (PBMCs). PBMCs are nucleus-containing cells (predominantly lymphocytes and monocytes) isolated from human peripheral blood. We isolated PBMCs from buffy coats using a standard isolation protocol [23]. BG4-flow analysis was performed and BG4 signals quantified as before. Similar to HeLa cells (Fig. 1), BG4-incubated PBMCs revealed a significant shift in peak distribution in comparison to the antibody-free control (Fig. 4a, Additional file 4: Fig. S4a-b), but only 16% of the population showed a BG4 signal. This data indicated that G4 structures form only in a certain set of human blood cells. In order to support this finding, we performed IF in PBMC cells from a healthy donor and detected G4 structures in BG4-incubated PBMCs (Fig. 4b). Further analysis with specific biomarkers will be required to address which subpopulation forms G4 structures.

In most cancer cells, an increase of G4 structures was detected [7, 8, 10, 11, 13]. It is assumed that the amount of G4 structures correlates with cancer progression and the mutagenic burden of the cells [24]. Leukemia is triggered by the abnormal proliferation of blood cells. Acute myeloid leukemia (AML) is the most common acute leukemia in adults. Several G4 ligands have been tested for the treatment of leukemia [25–28]. Interestingly, a bioinformatic analysis revealed that 70% of the genomic rearrangements in leukemia correlate with G4 motifs [29]. However, it is not known, if G4 structure formation is also enriched in AML cells. Therefore, we extended the previous analysis of blood cells and measured G4 levels in AML cells. We isolated PBMC from an AML patient with 99% of myeloid blasts in peripheral blood and performed BG-flow. PBMCs from a healthy donor served as a control. G4 structure levels in the AML cells were 2.17-fold lower than in healthy PBMC (Fig. 4c, d). Note, in this case, we already removed the background signal obtained in the negative control. This result needs further investigation to fully address its biological and possible clinical relevance. Note, AML PBMCs (here: 99% myeloid blasts) differ significantly from those of healthy individuals. It could therefore be that the observed decrease in G4 structures is not specific to recombination events in AML cells, but is based on different cell types. AML patients often have myeloid blasts, whereas healthy individuals have a heterogeneous composition of PBMCs (different lymphocyte subsets, monocytes, etc.). In summary, we could show that BG-flow is a fast and quantitative method to measure G4 structure levels in mixed cell populations of human blood cells.

### PDS induces a G4 structure increase in mouse macrophages

**Fig. 5.**
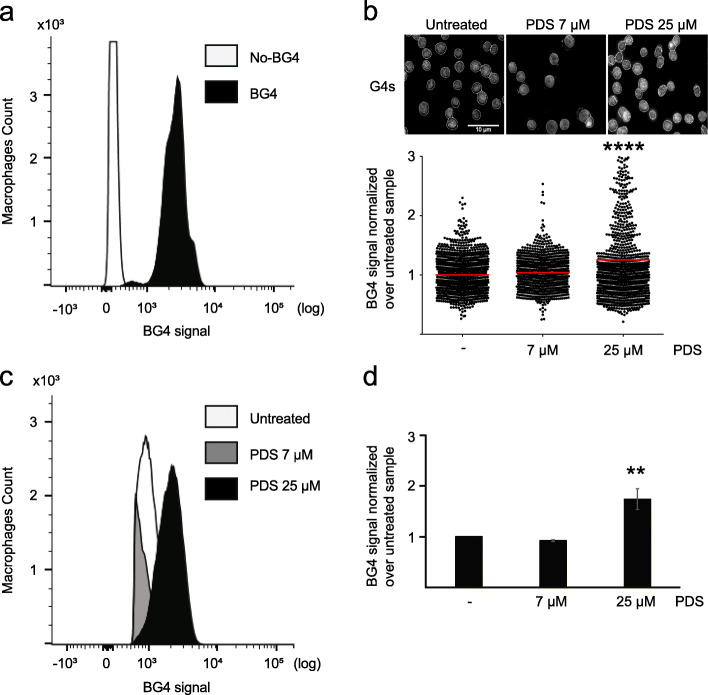
G4 flow cytometry analysis of mouse macrophage cells. **a** Histogram plot of the FL1-BG4 signal distribution in mouse macrophages incubated with BG4 (black) or without antibody (white). **b** Labeling of untreated mouse macrophages or cells incubated 24 h with 7 μM or 25 μM PDS with the BG4 antibody (white). Nucleus border is defined by the white line. Scale bar 10 μm. Below G4 levels determined by fluorescence intensity of nucleus normalized over untreated. Plotted results were based on the average of at least *n* = 3 biologically independent experiments. The horizontal gray line represents the mean value. Significance was determined using an ordinary one-way ANOVA multiple comparison: ****p < 0.0001. **c** Quantification of the FL1-BG4 signal in untreated mouse macrophages or cells incubated 4 h with 25 μM PDS. Plotted results were based on the average of *n* = 3 biologically independent experiments. Significance was calculated based on a one-sided Student’s *t*-test. **d** Histogram plot of the FL1-BG4 signal distribution in untreated mouse macrophages (white) or cells incubated 4 h with 25 μM PDS (black). Results are based on the average of at least *n* = 3 biologically independent experiments. Significance was calculated based on a one-sided Student’s *t*-test. Asterisks indicate statistical significance in comparison with wildtype cells: **p < 0.01. An excel file providing individual data values is available in the section “Availability of data and materials”

We successfully applied flow cytometry-based analysis of G4 structures in HeLa and THP-1 cells as well as PBMCs. We extended our analysis also to other species and examined murine macrophages. Mouse macrophages derived from the bone marrow, spleen, and peritoneum are routinely used in the research of the innate and adaptive immunity [30]. For our analysis, we used an immortalized mouse macrophage cell line [31]. In detail, the macrophages were isolated from mouse tibiae and immortalized by using SV40 virus transformation. We determined G4 structure levels in these cells in comparison to the antibody-free control. A shift of the BG4-signal was observed in BG4-incubated cells as compared to the antibody-free control (Fig. 5a, Additional file 5: Fig. S5a). As with human cells, these results only account for the BG4 signal from the nuclei. We added analogous to our previous studies PDS to the cells to control the specificity of the signal for G4 structures. The working concentration of PDS was assessed by MTT assay. A 4-h treatment with 25 μM PDS led to more than 90% viable cells (Fig. S5b). To monitor the effect of PDS on G4 structure levels, the cells were incubated with 7 and 25 μM PDS for 4 h. First, we performed standard IF analysis, which revealed a 1.4-fold increase in BG4 signal upon 25 μM PDS and no change for the 7 μM PDS in comparison to untreated cells (Fig. 5b). The BG-flow approach led to a fold increase of ~ 1.7-fold, whereas 7 μM PDS led to a 0.95-fold change compared to the untreated control (Fig. 5c, d, Additional file 5: Fig.S5a, c). Taken together, these results confirmed the reproducibility of BG-flow also in mouse cells.

## Discussion

Several publications demonstrated the significant binding of the single-chain antibody BG4 to G4 structures in vitro and in vivo [7, 8, 10, 13, 32]. Here, we reported a new protocol to use BG4 in a flow cytometry-based approach to detect G4 structures (BG-flow). Although BG4 and other G4 structure-specific antibodies were used in multiple publications [8, 10, 11, 13, 32], their use raised the concern that the antibody itself might induce the formation of G4 structures [33]. The here presented differences of G4 structure levels throughout the cell cycle and between different cell types argue for a biological effect rather than a BG4-driven stimulation of G4 structure formation (Figs. 2 and 3).

## Conclusions

BG-flow proved to be reproducible and consistent with established IF microscopy approaches. BG-flow cannot provide spatial information on the G4 structure localization unlike IF and ChIP-seq, but it bears some major advantages in terms of time and cost efficiency and flexibility. The whole protocol can be completed in 2 days and can be easily used also for detached or suspension cells (e.g., after stress or blood cells). Furthermore, BG-flow allows co-staining experiments to distinguish G4 patterns in different cell populations. BG-flow is a practical tool for the assessment of G4 structures in basic research as well as diagnostic applications, as demonstrated by our assessment of G4 structures in AML patient samples.

## Methods

### BG-flow

The protocol consists of three main steps: (i) trypsinization (for adherent cells), (ii) cell fixation and permeabilization, and (iii) blocking and incubation with antibodies. Secondary and tertiary antibodies were used to amplify the signal and make BG-flow more sensitive. The cell number used for each experiment is provided in the cell culture method section.

#### Trypsinization

The trypsinization step is mandatory for detaching adherent cells. The cells were washed once with PBS pH 7.4 to remove the culture media and then incubated at 37 °C for 3 min with trypsin-EDTA solution. Cells were resuspended in culture media and transferred into a 15-ml tube. Cells were collected by centrifugation at 200*g* at room temperature (RT) for 5 min.

#### Fixation and permeabilization

The cell pellet was resuspended in 1 ml 50% DMEM and 50% methanol/acetic acid (3:1), transferred into a 1.5-ml tube, and incubated for 5 min at RT. The cells were centrifuged for 5 min at 300*g* (RT) and the supernatant discarded. Fixation was performed by incubating in 3:1 (v/v) methanol/acetic acid solution for 10 min at RT. Alternatively, the cells were fixed by resuspending the pellet in 2% (v/v) PFA in PBS for 15 min at RT. Fixed cells were washed twice with PBS pH 7.4. Permeabilization was performed with 0.1% (v/v) Triton X-100 in PBS pH 7.4 for 5 min at RT. Cells were washed twice with PBS pH 7.4 for 10 min at RT in a tube rotator (30 rpm). After each wash step, the cells were centrifuged 5 min at 300*g* (RT) and the supernatant removed.

#### Blocking, antibody incubation, and flow cytometry information

Blocking was performed with 2% (w/v) nonfat dry milk in PBS pH 7.4 (blocking buffer) for 45 min at RT in a tube rotator (30 rpm). Blocked cells were incubated with 5 μg of BG4 diluted in blocking buffer for 2 h at RT in a tube rotator (30 rpm). Cells were washed twice with 0.1% (v/v) Tween in PBS pH 7.4 for 10 min at RT in a tube rotator (30 rpm). After each wash step, the cells were centrifuged for 5 min at 300*g* (RT) and the supernatant removed.

BG4 is a single-chain antibody containing three FLAG tags (DYKDDDDK epitope). For signal amplification, the cells were incubated with a rabbit antibody against the DYKDDDDK epitope (Cell Signaling ref #2368) diluted 1:250 in blocking buffer solution for 1 h at RT in a tube rotator (30 rpm). Cells were then washed twice with 0.1% (v/v) Tween in PBS pH 7.4 for 10 min at RT in a tube rotator (30 rpm). After each step, the cells were centrifuged 5 min at 300*g* (RT) and the supernatant disposed.

Finally, the cells were incubated with a fluorescent secondary antibody (Alexa Fluor® 488—Invitrogen ref #A11008) diluted 1:600 in blocking buffer solution for 1 h at RT in a tube rotator (30 rpm). Cells were then washed once with 0.1% Tween in PBS pH 7.4 and once with PBS pH 7.4 for 10 min at RT in a tube rotator (30 rpm). After each wash step, the cells were centrifuged 5 min at 300*g* (RT) and the supernatant disposed.

In indicated experiments, the cells were co-stained with 10 μg ml^−1^ DAPI solution (alternatively, the staining could be performed with 50 μg ml^−1^ PI solution or 1.2 μg ml^−1^ Hoechst33258 solution) in PBS pH 7.4, for 30 min at 37 °C. The quality of the staining could be increased by a co-treatment with 50 μg ml^− 1^ RNase A.

Cells were finally resuspended in 1 ml PBS pH 7.4 and analyzed by flow cytometry on a BD FACSCanto™ II Cell Analyzer. After data acquisition, data was analyzed using FlowJo [19] gating the cell for the size (forward scatter (FSC)) and granularity of the cells (side scatter (SSC)). A pool of samples not incubated with BG4 was used as a negative control.

### Cell lines and culture conditions

HeLa and THP-1 cells were purchased from ATCC. Mouse macrophages and MCF-7 were kindly provided by the Abdullah and Feldmann lab (both University Hospital Bonn), respectively. HeLa, MCF-7, and mouse macrophages were grown in glutamine-rich DMEM (Gibco™) supplemented with 10% fetal bovine serum (FBS, Gibco™). THP-1 cells were grown in glutamine-rich RPMI (Gibco™) supplemented with 10% FBS. All cell lines were passaged 2–3 times a week and incubated at 37 °C in 5% CO_2_. For HeLa and THP-1 cells, 1.2–1.5 × 10^6^ cells were seeded in 10-cm dishes, whereas 2.2–2.5 × 10^6^ were seeded for mouse macrophages. PDS and PhenDC_3_ treatments were performed at 60% confluence. For PBMC, 5 × 10^6^ of cells extracted by buffy coat were used for the analysis.

All the cell lines used for the experiments were tested for mycoplasma contamination.

### BG4 purification

The plasmid expressing the single-chain antibody specific to G4 structures (BG4) was kindly provided by S. Balasubramanian (University of Cambridge, UK). BL21(DE3) competent cells containing BG4 plasmid were grown in 2xTY media (1.6% bacto tryptone, 1% bacto yeast extract, 0.5% NaCl) 1% glucose and 50 μg ml^−1^ kanamycin. The overnight culture was expanded in 2 l of 2xTY media containing 50 μg ml^−1^ kanamycin. BG4 expression was induced with the addition of 0.5 mM IPTG (isopropyl-β-D-thiogalactopyranoside) and incubation at 25 °C, 220 rpm, overnight. Bacterial cells were lysed in TES buffer (50 mM Tris-Cl pH 8.0, 1 mM EDTA, 20% sucrose) on ice for 10 min. The lysate was diluted 1:5 with distillated water and left on ice for 15 min prior to centrifugation for 30 min at 16,000*g* (4 °C). The supernatant was filtered (0.45 μm) and BG4 was purified on a Ni-NTA sepharose column (GE healthcare) pre-equilibrated with TES buffer. The column was washed twice with one column volume PBS supplemented with 100 mM NaCl and 10 mM imidazole (pH 8.0). BG4 was eluted with PBS supplemented with 250 mM imidazole (pH 8.0). The elution buffer was exchanged with inner cell salt buffer (25 mM Hepes (pH 7.6), 110 mM KCl, 10.5 mM NaCl, 1 mM MgCl_2_). BG4 was concentrated using an Amicon Ultra-15 Centrifugal Filter Unit with 10-kDa cutoff (Millipore). BG4 was quantified by NanoDrop and Qubit (Thermo Scientific) and stored at − 80 °C. Purity of the BG4 preparation was monitored by SDS-PAGE.

### BG4 immunofluorescence

Cells were seeded in 24-multiwell plates (HeLa 4 × 10^4^, THP-1 3 × 10^4^, mouse macrophages 7 × 10^4^). Twenty-four hours post-seeding, cells were incubated with PDS for 4 h (mouse macrophages) or 24 h (HeLa, THP-1). Cells were pre-fixed with a solution of 50% DMEM and 50% methanol/acetic acid (3:1) at RT for 5 min. After a brief wash with methanol/acetic acid (3:1), the cells were fixed with methanol/acetic acid (3:1) at RT for 10 min. Cells were then permeabilized with 0.1% (v/v) Triton X-100 in PBS at RT for 3 min under gentle rocking and exposed to the blocking solution (2% (w/v) nonfat dry milk in PBS, pH 7.4) for 1 h at RT under gentle rocking. Two micrograms of BG4 antibody in blocking solution was used per slide (2 h at RT). Cells were then incubated with a rabbit antibody against the DYKDDDDK epitope (Cell Signaling ref #2368) diluted 1:800 in blocking solution for 1 h under gentle rocking at RT. Next, cells were incubated at RT with Cyanine 3 goat anti-rabbit IgG (Life technologies ref #A10520) diluted 1:1000 in blocking solution for 1 h at RT under gentle rocking. After each step, cells were washed three times for 10 min with 0.1% (v/v) Tween-20 in PBS under gentle rocking. The cover glasses were mounted with Fluoroshield mounting media (Merck) containing DAPI (for nuclear staining).

Slides were visualized at RT by using a fluorescence microscope (Zeiss Axio Observer Z1). Fluorescence signal was determined using integrated density obtained by Fiji [34]. The plots were prepared using GraphPad Prism 6.

### PBMC extraction

Buffy coats for human PBMC cells were obtained from voluntary blood donors at the University Hospital Bonn. Briefly, whole blood samples, diluted 1:6 in PBS pH 7.4, were centrifuged at 350*g* for 15 min without a break. After centrifugation, there are three layers present: from top to bottom: (i) plasma, containing cell-free DNA; (ii) “buffy coat,” containing PBMC cells; and (iii) red blood, containing erythrocytes. PBMC cells were separated and washed twice with PBS pH 7.4. After each wash, cells were centrifuged at 300*g* for 5 min (RT).

### Cell survival—MTT assay

Cytotoxicity of PDS was determined with a MTT assay in THP-1 and mouse macrophages. A MTT assay is a colorimetric method that measures mitochondrial reductive function and is useful as an indicator of cell death or growth inhibition. Seeding was performed in 96-well plates. After 4- or 24-h incubation with PDS, the cells were washed with PBS and fresh medium containing 500 μg ml^−1^ of Thiazolyl Blue Tetrazolium Bromide solution (Sigma) was added to each well and incubated for 4 h in an incubator at 37 °C in 5% CO_2_. The medium was subsequently removed, and precipitated formazan crystals were solubilized in 100 μl dimethylsulfoxide (DMSO). Absorbance at 570 nm was measured using a multiplate reader. Cell survival directly correlated with the absorbance values at 570 nm. Absorbance was then normalized against untreated cells (negative control) and used to obtain a compound concentration with a cell viability ≥ 80%.

### Statistical significance and figure preparation

Statistical significance was determined by ordinary one-way ANOVA multiple comparison test for IF and one-sided Student’s *t*-test for FC. Figures were prepared in Adobe Illustrator.

## Supplementary Information

**Additional file 1: Figure S1.** BG-flow is suitable for HeLa cells. a) HeLa cells gated for granularity (side scatter – SSC) and size (forward scatter - FSC). b) Labeling of untreated HeLa cells or cells incubated 24 h with 1 μM or 10 μM PDS with the BG4 antibody (green) and DAPI (blue). Scale bar: 10 μm. c) Distribution plot of the BG4 signal in HeLa cells unstained with BG4 (red), untreated (cyan) or incubated 24 h with 1 μM (green) or 10 μM (orange) PDS. d) Histogram plot of the BG4 signal in untreated HeLa cells (white) and cells incubated 24 h with 1 μM (gray) or 10 μM (black) PDS and fixed with PFA. **e)** Histogram plot of the BG4 signal in HeLa cells, fixed with PFA, unstained with BG4 (white), untreated (gray) or incubated 24 h with 10 μM PDS (black).

**Additional file 2: Figure S2.** Human monocytes showed increased G4 levels after PDS treatment. a) THP-1 cells gated for granularity (side scatter – SSC) and size (forward scatter - FSC) b) Cell vitality determined by MTT assay in THP-1 cells treated with different concentration of PDS. Graph shows the % of vitality compared to untreated control (100%). Average of *N* = 3 biologically independent experiments is plotted ± SD **c)** Distribution plot of the FL1-BG4 signal in THP-1 cells unstained with BG4 (red), untreated (cyan) or treated 24 h with 25 μM (green) or 50 μM (orange) PDS.

**Additional file 3: Figure S3**. MCF-7 cells showed higher BG4 signal in S/G2 phase. a) MCF-7 cells gated for granularity (side scatter – SSC) and size (forward scatter – FSC). b) Distribution plot of the BG4 signal in MCF-7 cells unstained (red) or stained with BG4 (cyan).

**Additional file 4: Figure S4**. BG-Flow is suitable for Human PBMCs. a) PBMC Cells gated for granularity (side scatter – SSC) and size (forward scatter - FSC) b) Distribution plot of the BG4 signal in PBMCs extracted from an AML patient, unstained (red) or stained with BG4 (cyan).

**Additional file 5: Figure S5.** BG-Flow is suitable for mouse macrophages. a) Mouse macrophages gated for granularity (side scatter – SSC) and size (forward scatter - FSC). b) Cell vitality as determined by a MTT assay in mouse macrophages treated with different concentration of PDS. Graph shows the % of vitality compared to untreated control (100%). Average of *n* = 3 biologically independent experiments are plotted ± SD. **c)** Distribution plot of the FL1-BG4 signal in macrophages unstained with BG4 (red), untreated (cyan) or treated 4 h with 7 μM (green) or 25 μM (orange) PDS.

**Additional file 6.** Raw data for Figures 1-5.

## Data Availability

All data generated or analyzed during this study are included in this published article and its supplementary information files. Raw data for Figs. 1, 2, 3, 4, and 5 can be found in Additional file 6.

## Abbreviations

FC: Flow cytometry
IF: Immunofluorescence
SSC: Side scatter
FSC: Forward scatter
ChIP-seq: Chromatin immunoprecipitation and sequencing
G4: G-quadruplex
MFI: Mean fluorescence intensity
PFA: Paraformaldehyde
PDS: Pyridostatin
