# Supplementary figures and images for: BG-flow, a new flow cytometry tool for G-quadruplex quantification in fixed cells
Source: BMC Biol. 2021 Mar 11;19:45. doi: 10.1186/s12915-021-00986-6 (PMC7953821; doi:10.1186/s12915-021-00986-6)

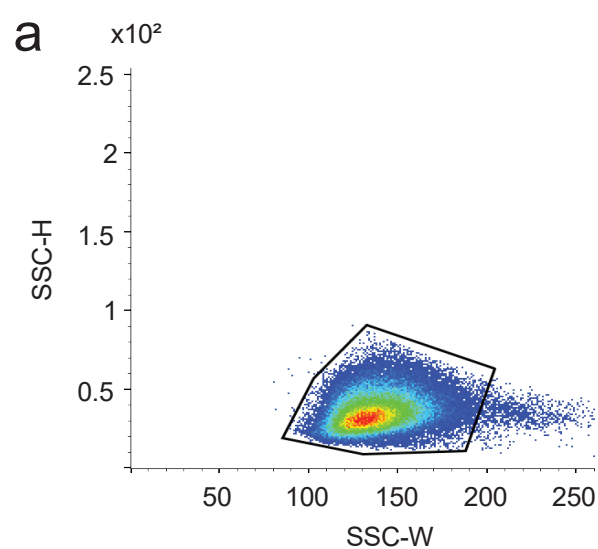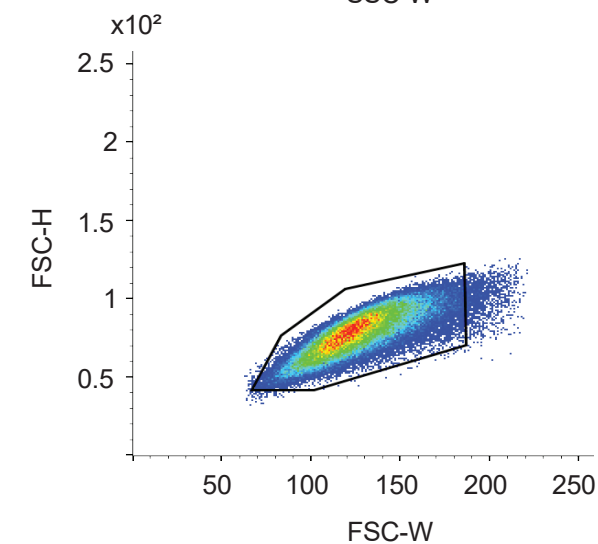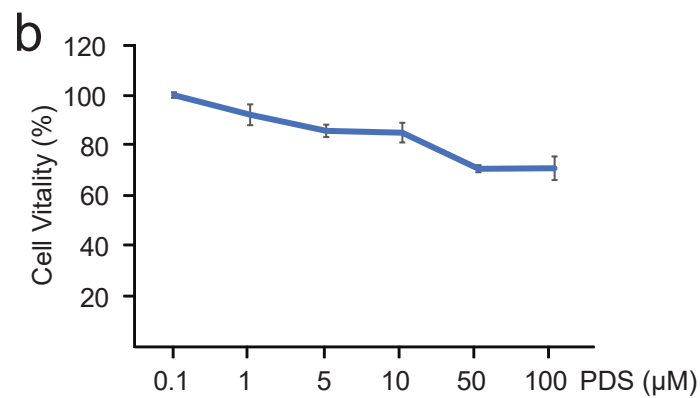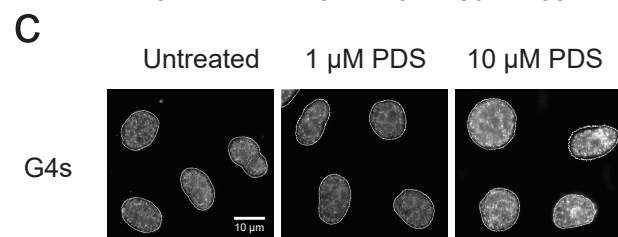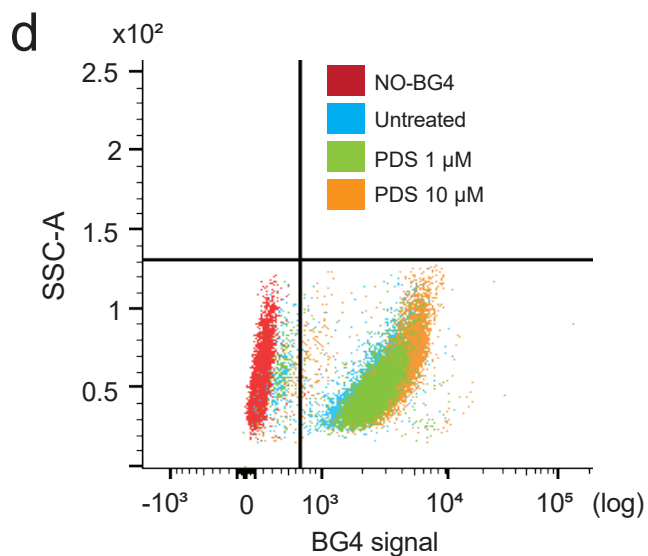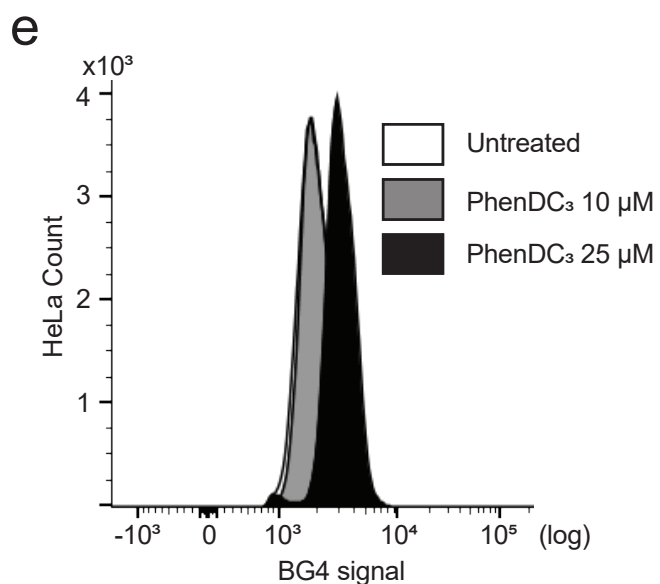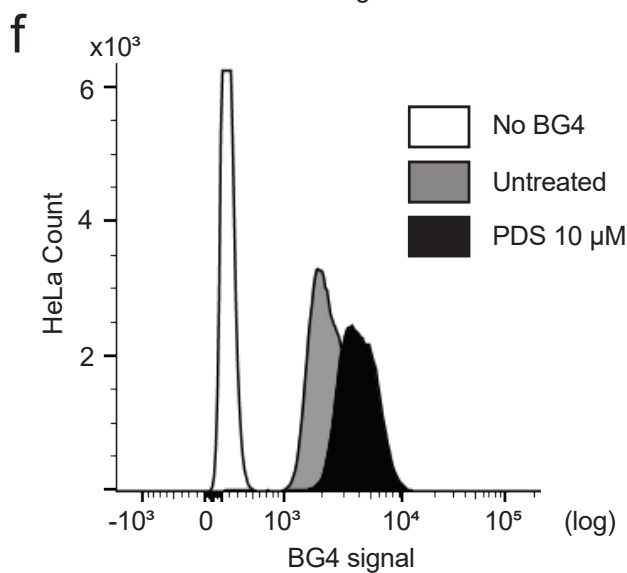

Supplement: Supplementary file 1 — Additional file 1: Figure S1. BG-flow is suitable for HeLa cells. a) HeLa cells gated for granularity (side scatter – SSC) and size (forward scatter - FSC). b) Labeling of untreated HeLa cells or cells incubated 24 h with 1 μM or 10 μM PDS with the BG4 antibody (green) and DAPI (blue). Scale bar: 10 μm. c) Distribution plot of the BG4 signal in HeLa cells unstained with BG4 (red), untreated (cyan) or incubated 24 h with 1 μM (green) or 10 μM (orange) PDS. d) Histogram plot of the BG4 signal in untreated HeLa cells (white) and cells incubated 24 h with 1 μM (gray) or 10 μM (black) PDS and fixed with PFA. e) Histogram plot of the BG4 signal in HeLa cells, fixed with PFA, unstained with BG4 (white), untreated (gray) or incubated 24 h with 10 μM PDS (black). [file 12915_2021_986_MOESM1_ESM.pdf]

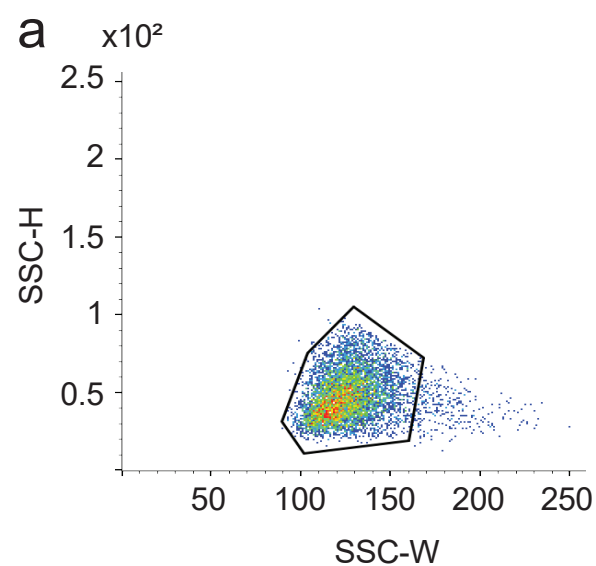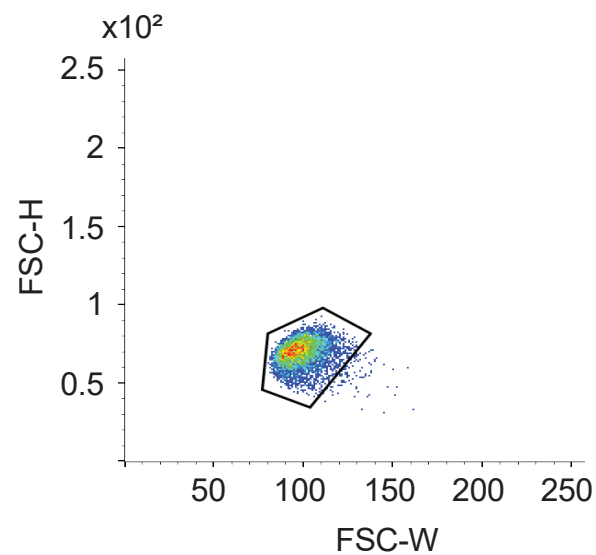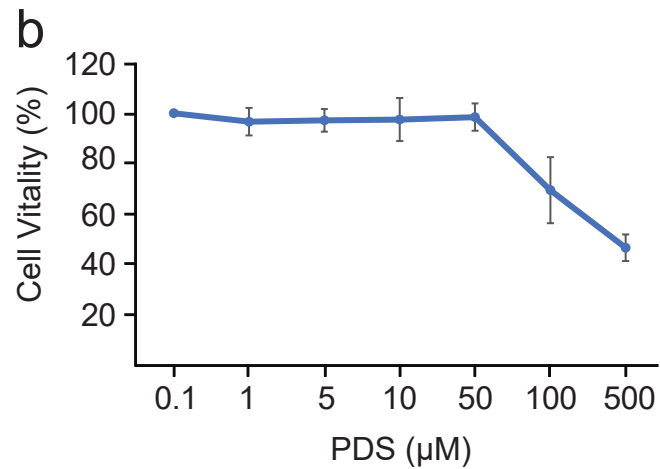

80% Viability =  $\sim 75 \mu\text{M}$

IC50 =  $463.5 \pm 2.5 \mu\text{M}$

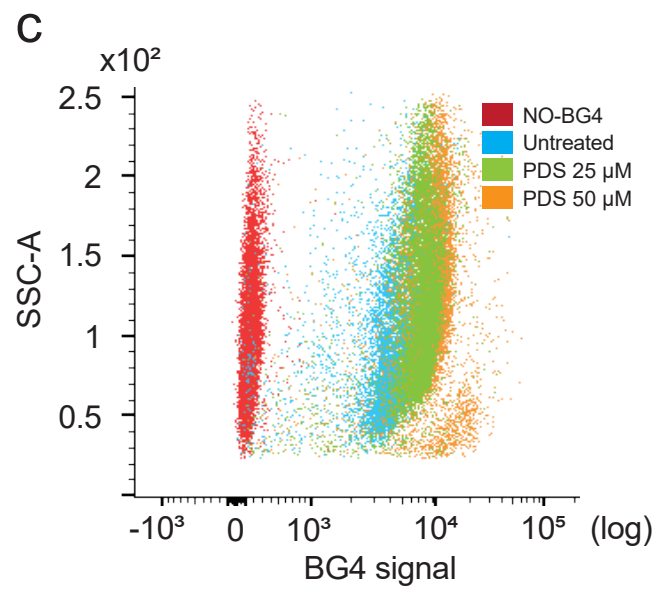

Supplement: Supplementary file 2 — Additional file 2: Figure S2. Human monocytes showed increased G4 levels after PDS treatment. a) THP-1 cells gated for granularity (side scatter – SSC) and size (forward scatter - FSC) b) Cell vitality determined by MTT assay in THP-1 cells treated with different concentration of PDS. Graph shows the % of vitality compared to untreated control (100%). Average of N = 3 biologically independent experiments is plotted ± SD c) Distribution plot of the FL1-BG4 signal in THP-1 cells unstained with BG4 (red), untreated (cyan) or treated 24 h with 25 μM (green) or 50 μM (orange) PDS. [file 12915_2021_986_MOESM2_ESM.pdf]

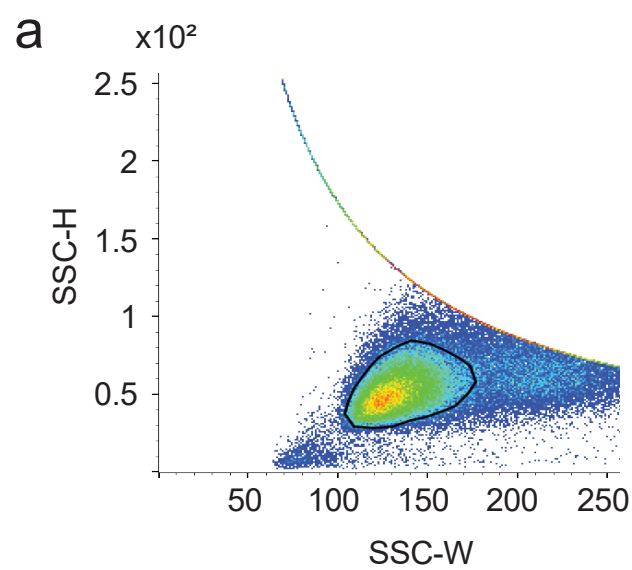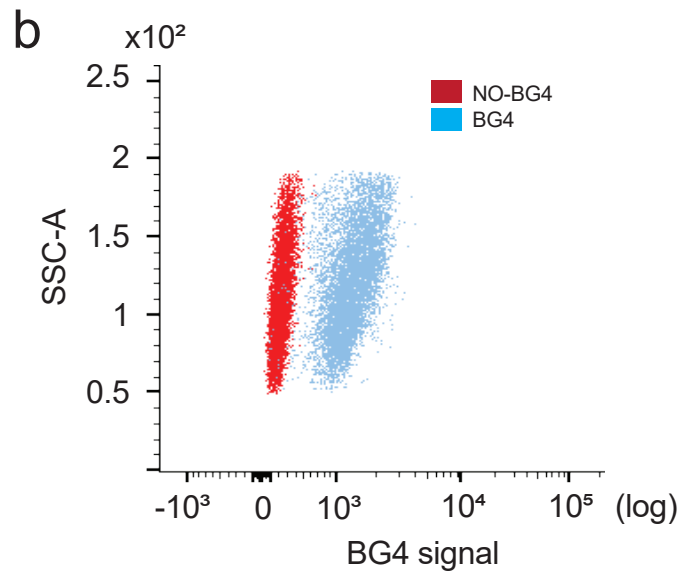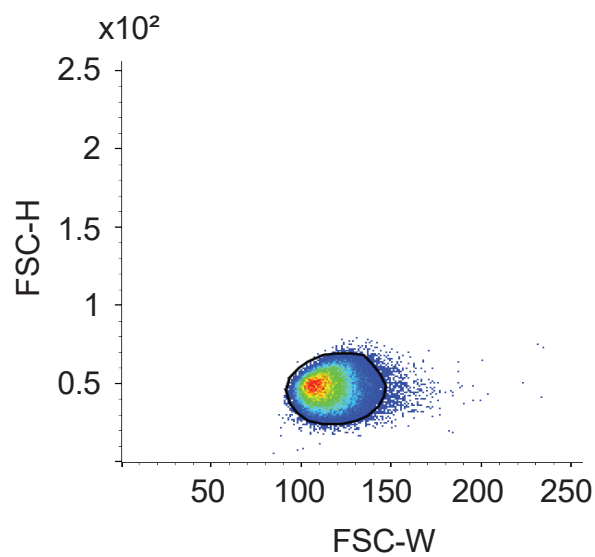

Supplement: Supplementary file 3 — Additional file 3: Figure S3. MCF-7 cells showed higher BG4 signal in S/G2 phase. a) MCF-7 cells gated for granularity (side scatter – SSC) and size (forward scatter – FSC). b) Distribution plot of the BG4 signal in MCF-7 cells unstained (red) or stained with BG4 (cyan). [file 12915_2021_986_MOESM3_ESM.pdf]

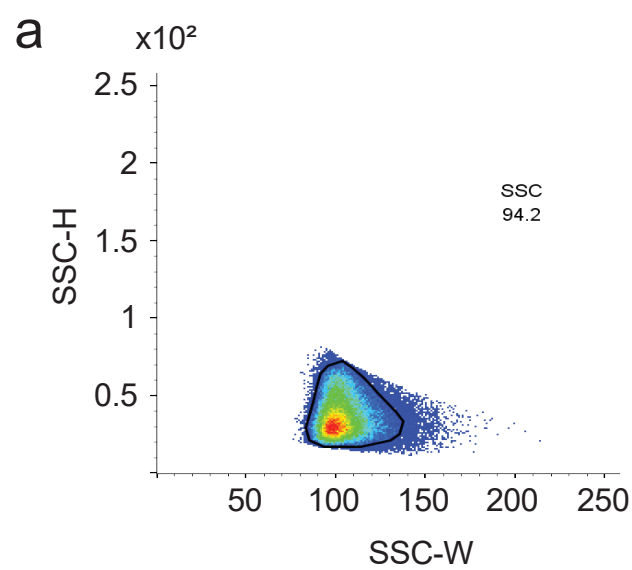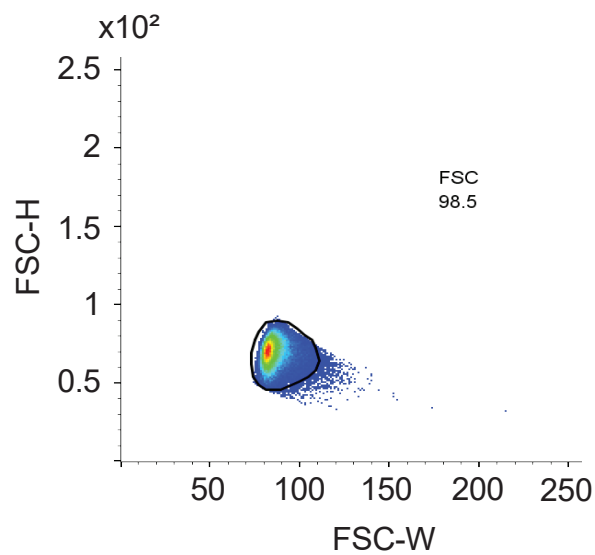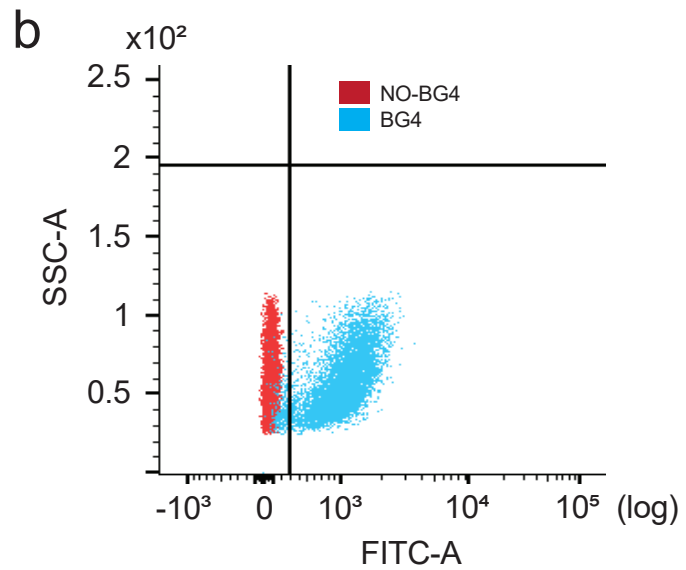

Supplement: Supplementary file 4 — Additional file 4: Figure S4. BG-Flow is suitable for Human PBMCs. a) PBMC Cells gated for granularity (side scatter – SSC) and size (forward scatter - FSC) b) Distribution plot of the BG4 signal in PBMCs extracted from an AML patient, unstained (red) or stained with BG4 (cyan). [file 12915_2021_986_MOESM4_ESM.pdf]

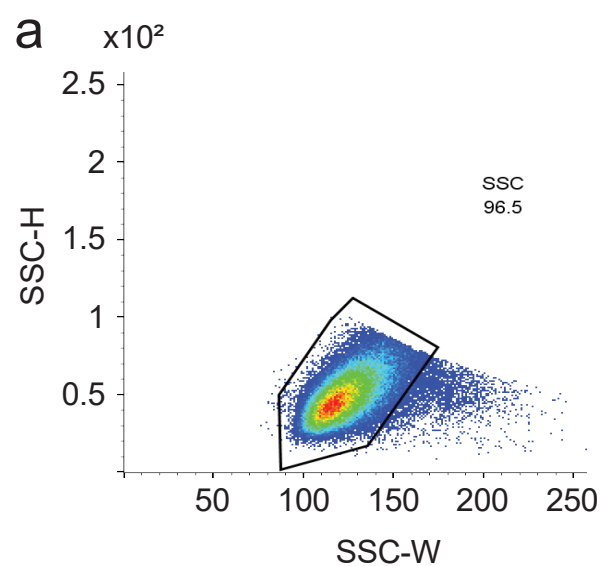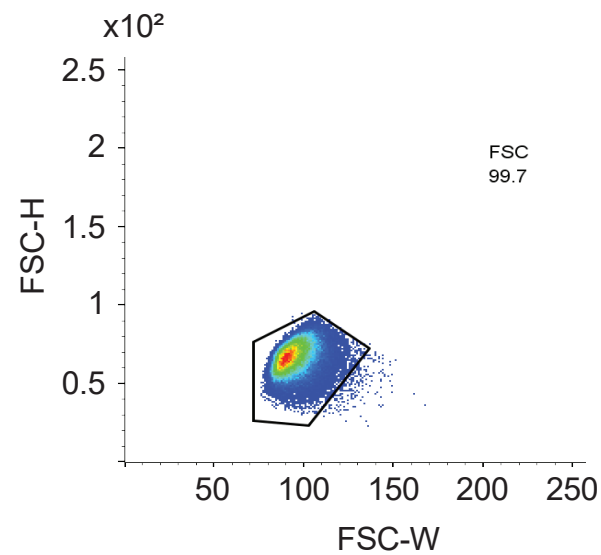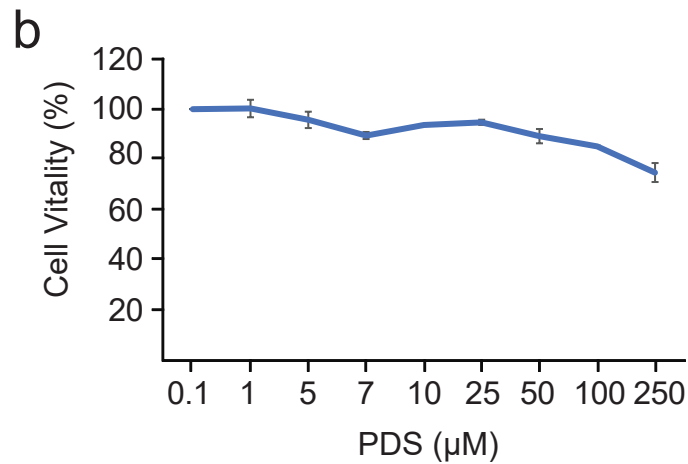

80% Viability =  $\sim 250 \mu\text{M}$

IC50 =  $> 500 \mu\text{M}$

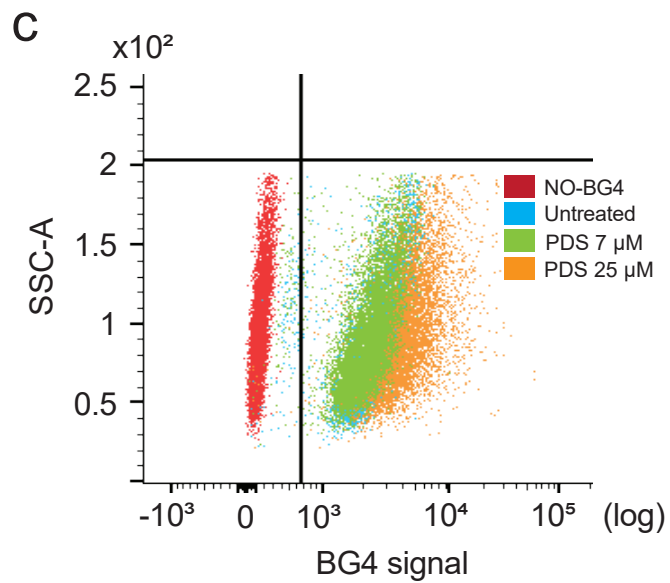

Supplement: Supplementary file 5 — Additional file 5: Figure S5. BG-Flow is suitable for mouse macrophages. a) Mouse macrophages gated for granularity (side scatter – SSC) and size (forward scatter - FSC). b) Cell vitality as determined by a MTT assay in mouse macrophages treated with different concentration of PDS. Graph shows the % of vitality compared to untreated control (100%). Average of n = 3 biologically independent experiments are plotted ± SD. c) Distribution plot of the FL1-BG4 signal in macrophages unstained with BG4 (red), untreated (cyan) or treated 4 h with 7 μM (green) or 25 μM (orange) PDS. [file 12915_2021_986_MOESM5_ESM.pdf]
